# Mitigation effect of alpha-tocopherol and thermo-priming in *Brassica napus* L. under induced mercuric chloride stress

**DOI:** 10.1186/s12870-024-04767-5

**Published:** 2024-02-13

**Authors:** Fazal Amin, Arwa Abdulkreem AL-Huqail, Sami Ullah, Muhammad Nauman Khan, Alevcan Kaplan, Baber Ali, Majid Iqbal, Fahmy Gad Elsaid, Sezai Ercisli, Tabarak Malik, Sami Asir Al-Robai, Amany H. A. Abeed

**Affiliations:** 1 Department of Botany, University of Peshawar, Peshawar, 25120 Pakistan; 2 Department of Biology, College of Science, Princess Nourah bint Abdulrahman University, P.O. Box 84428, Riyadh, 11671 Saudi Arabia; 3 Department of Botany, Islamia College, Peshawar, 25120 Pakistan; 4 Biology Laboratory, University Public School, University of Peshawar, Peshawar, 25120 Pakistan; 5 Department of Crop and Animal Production, Sason Vocational School, Batman University, Batman, 72060 Turkey; 6 Department of Plant Sciences, Quaid-I-Azam University, Islamabad, 45320 Pakistan; 7 Institute of Geographic Sciences and Natural Resources Research, University of Chinese Academy of Sciences, Beijing, 100040 China; 8 Biology Department, College of Science, King Khalid University, 61421 Abha, Al-Faraa, Asir Saudi Arabia; 9 Department of Horticulture, Agricultural Faculty, Ataturk University, Erzurum, 25240 Turkey; 10 Department of Biomedical Sciences, Institute of Health, Jimma University, 378 Jimma, Ethiopia; 11 Department of Biology, Faculty of Science, Al-Baha University, 1988 Al-Baha, Saudi Arabia; 12 Department of Botany and Microbiology, Faculty of Science, Assiut University, Assiut, 71516 Egypt

**Keywords:** Antioxidant enzymes, *Brassica napus* L., Chlorophyll, Heavy metal, Osmolytes

## Abstract

Soil pollution with heavy metals has grown to be a big hassle, leading to the loss in farming production particularly in developing countries like Pakistan, where no proper channel is present for irrigation and extraction of these toxic heavy metals. The present study aims to ameliorate the damages caused by heavy metal ions (Hg-Mercury) on rapeseed (*Brassica napus* L.) via a growth regulator (α-tocopherol 150 mg/L) and thermopriming technique at 4 °C and 50 °C to maintain plant agronomical and physiological characteristics. In pot experiments, we designed total of 11 treatments viz.( T0 (control), T1 (Hg4ppm), T2 (Hg8ppm), T3 (Hg4ppm + 4 °C), T4 (Hg4ppm + 4 °C + tocopherol (150 m/L)), T5 (Hg4ppm + 50 °C), T6 (Hg4ppm + 50 °C + tocopherol (150 mg/L)), T7 (Hg8ppm + 4 °C), T8 (Hg8ppm + 4 °C + tocopherol (150 mg/L)), T9 (Hg8ppm + 50 °C), T10 (Hg8ppm + 50 °C + tocopherol (150 mg/L) the results revealed that chlorophyll content at *p* < 0.05 with growth regulator and antioxidant enzymes such as catalase, peroxidase, and malondialdehyde enhanced up to the maximum level at T5 = Hg4ppm + 50 °C (50 °C thermopriming under 4 ppm mercuric chloride stress), suggesting that high temperature initiate the antioxidant system to reduce photosystem damage. However, protein, proline, superoxide dismutase at *p* < 0.05, and carotenoid, soluble sugar, and ascorbate peroxidase were increased non-significantly (*p* > 0.05) 50 °C thermopriming under 8 ppm high mercuric chloride stress (T9 = Hg8ppm + 50 °C) representing the tolerance of selected specie by synthesizing osmolytes to resist oxidation mechanism. Furthermore, reduction in % MC (moisture content) is easily improved with foliar application of α-tocopherol and 50 °C thermopriming and 4 ppm heavy metal stress at T6 = Hg4ppm + 50 °C + α-tocopherol (150 mg/L), with a remarkable increase in plant vigor and germination energy. It has resulted that the inhibitory effect of only lower concentration (4 ppm) of heavy metal stress was ameliorated by exogenous application of α-tocopherol and thermopriming technique by synthesizing high levels of proline and antioxidant activities in maintaining seedling growth and development on heavy metal contaminated soil.

## Introduction

Climate change as well as rising temperatures are a big concern in many parts of the globe [1, 2]. The main environmental factor affecting vegetation worldwide has been determined to be climate change [3, 4]. Plant development and production are impacted by environmental changes such as temperature rise, changes in carbon dioxide (CO_2_) levels, patterns of precipitation, and related abiotic stressors like heat, drought [5], salt [6], and the state of the soil's nutrients [7, 8]. Through an examination of the results from the IPCC's (Intergovernmental Panel for Climate Change) Assessment Report 4 (AR4) model ensemble, we are able to show that rising temperatures in the Amazon and increased water stress are both expected to occur in the twenty-first century [9–11]. Similarly, an increase in temperature from 3 to 4 °C will lead to a decrease in agricultural crop production of up to 15–35% in Asia and Africa and 25–35% in the Middle East [1]. Minor temperature changes can lead to the expression or suppression of hundreds of genes in plants that lead to a decline in crop production [12]. Stress in plants is formerly related to stress resistance, which is the ability or fitness of a plant to live under unfavorable conditions [13].

Due to uneven substance usage in the past ten years, Pakistan has experienced extreme environmental stress [14], particularly in the form of soil stress. As a result, typically dormant stress-related genes are rapidly activated by environmental stress [15]. Plant interactions with environmental stress factors are known to contribute to the initiation of various defense mechanisms [16, 17], resulting in qualitative and/or quantitative improvements in the development of plant metabolites, activation of hormone signaling pathways regulated by abscisic acid (ABA), salicylic acid, jasmonic acid, and ethylene, as well as reactive oxygen species (ROS) signaling pathways [18]. Furthermore, Abiotic stresses include cold, dryness, salt, and heavy metals mostly affect plant development and agricultural yield [16]. Plants can initiate a variety of genetic, cellular, and physiological modifications in response to abiotic stress. [19, 20]. Heavy metals are elements with a large atomic mass and are at least five times denser than water [21]. Heavy metal stress has become a major concern in many terrestrial ecosystems across the world [22]. By collecting heavy metals, modern industrialization has a negative impact on soil as well as crop yield. Heavy metals including Zn, Cu, Mo, Mn, Co, Hg, and Ni are required for critical biological activities and developmental pathways [23–26]. Heavy metals have a high ecological significance due to their persistence, toxicity, and bioaccumulation capability, and an excess of heavy metals in soil affects plant productivity and yield greatly [27, 28]. Mercury (Hg) is an important metal because of its use in multiple ways, e.g. Hg^2+^, HgS, HgO, and methyl-Hg, respectively. Mercury is ubiquitous, being found in all ecospheres as well as igneous rocks in trace quantities. Growing research has shown that Hg^2+^ can be stored freely in advanced and aquatic plants [17].

Due to mercury’s high toxicity and frequent prevalence, mercury poisoning (Hg) has drawn particular interest [29]. In the soil and water, the predominant form of mercury is Hg^2+^. Because of its reactive characteristics, mercury is particularly phytotoxic [30]. Reduced nutrient absorption is a further toxic result of the accumulation of Hg^2+^ in plants and reduced photosynthesis [31]. Soil and water polluted with radioactive metals pose a significant danger to environmental welfare and food safety. Lead (Pb), mercury (Hg), and cadmium (Cd) are examples of non-essential metals that have no significant biological function. Rather, when these metals are present in larger concentrations, they interact with biological, systemic, and metabolic systems [32]. Soil ecosystems are contaminated with heavy metals by human-induced activities [33]. A hazardous concentration of heavy metals in agricultural soils is unknown [34]. Heavy metal contamination is a severe concern in Pakistan, rapidly depleting soil, water, and food resources owing to inattention [35]. Therefore the status of heavy metal in the study area district Peshawar, Pakistan was described by Amin and Ahmad, [35]. They reported different heavy metal contents showed ppm ranges of: Cr = 0.06–3.2, Co = 0.3–2.4, Ni = 0.17–5.97, Cu = 0.88–8.8, Zn = 0.81–17, Fe = 3–57, and Pb = 2–25. Heavy metals, particularly mercury, are environmental contaminants. Heavy metals that accumulate to hazardous amounts in agricultural soils have a negative impact on crop health and yield [36].

Seed priming is a pre-sowing seed preparation that allows seeds to be moderately hydrated in order to ingest water and pass through the early stages of germination, allowing seeds to germinate more effectively [37–39]. Seed priming has been successfully demonstrated to enhance germination and emergence, especially under stress conditions, in seeds of many crops [40]. Seed priming involves pre-soaking seeds in distilled water or osmotic solutions. Seed priming is a simple, cost-effective, and persuasive method for improving seed germination, early seedling growth, and production under normal and stressful conditions [41, 42]. Thermopriming is a seed pre-treatment method that involves exposing a seed to a given temperature for a set period of time to evaluate the temperature influence on the coat, physiology, and growth of the seed [43, 44]. To accelerate the germination and seedling development in crops under natural and saline conditions, several seed priming treatments have been used. Priming appears to be a feasible technology for high vigor, followed by better yields in certain crops for quick and uniform emergence [45]. This, together with emergence synchronisation, reduces the time between seed sowing and seedling emergence [46]. In terms of plant development, yield quantity, and efficiency, the use of antioxidants such as vitamins has received a lot of attention in reducing the negative effects of water and salinity stress on plants. Alpha-tocopherol (α-t), so-called vitamin E, is a lipophilic antioxidant with a low molecular weight that usually prevents plants from cellular oxidation caused by stress. It is well known that α-tocopherol is used exogenously to enhance plant growth and production processes under unfavorable environmental conditions [47–49].

After palm and soyabean oil, rapeseed (*B. napus* L.) is the third largest oilseed crop in the world, accounting for nearly 16% of the entire supply of vegetable oil worldwide. Currently, most commercial rapeseed cultivars have brown to black seed color. Previous studies have shown that *B. napus* has yellow seeds. *B. napus* has a thinner seed coat, a reduced proportion of pigment and hull, and a higher content of oil and protein than the black seed form. With these superior characteristics, yellow seed is generally recognized as a premium attribute and is a global subject of rapeseed science [50, 51].

The alpha-tocopherol and thermopriming will mitigate the effect of mercuric chloride toxicity in *B. napus*. It is expected that the uptake of mercury by the *Brassica* will be reduced in the presence of alpha-tocopherol and that the plants will be less affected by the mercury if they are thermoprimed. Looking into the current and future expected environmental pollution with mercury, the aim of the current research work has been planned to assess mercury's impact on canola crop growth responses, osmo-protection capacities, and antioxidant enzyme system under the effect of -tocopherol foliar spray and thermopriming treatment. Such studies have demonstrated its significance in the region, as Pakistan is a developing country with no effective waste recycling infrastructure to stabilise our agricultural growth rate. There is also a need to adopt rapid adaptive steps suggested by the latest and relevant results of other researchers. Heavy metals, particularly mercury, are environmental contaminants. Heavy metals that accumulate to hazardous amounts in agricultural soils have a negative impact on crop health and yield. Therefore, the present study was aimed to ameliorate the mitigation effect via different mitigation methods like priming and thermo priming under mercuric chloride stress in *B. napus* via a growth regulator and contributing to reducing environmental pollution, which reflects positively on maintaining food security to avoid hunger and poverty in the long term, and contributes to achieving sustainable development goals and raising the quality of life.

## Materials and methods

### Experimental design, study site description, and climate

The certified and disease-free seeds of *B. napus* L. variety (NIFA Gold) were procured from the Nuclear Institute of Food and Agriculture Peshawar (NIFA). The experiment was carried out in 2019 in the greenhouse at the University of Peshawar, Peshawar (latitude, 34.01’ N, longitude 71.48’ E, and altitude of 1199 feet) weather ranges between 5 °C (in January) and 39 °C (in June) with a mean annual rainfall of about 513 mm under natural conditions during the canola growing season January 2019. Peshawar with its tropical climate lies in the Iranian plateau area [24, 52]. With a semi-arid climate, the district of Peshawar has very hot summers and mild winters [53]. A total of 33 pots in which 3 pots per treatment (*n* = 11) were used. In total 11 treatments (T0 (control), T1 (Hg4ppm), T2 (Hg8ppm), T3 (Hg4ppm + 4 °C), T4 (Hg4ppm + 4 °C + tocopherol (150 m/L)), T5 (Hg4ppm + 50 °C), T6 (Hg4ppm + 50 °C + tocopherol(150 mg/L)), T7 (Hg8ppm + 4 °C), T8 (Hg8ppm + 4 °C + tocopherol(150 mg/L)), T9 (Hg8ppm + 50 °C), T10 (Hg8ppm + 50 °C + tocopherol(150 mg/L))) were designed (in triplicate) in a complete randomized block design (CRBD). Ten seeds per pot were planted (18 cm lower inside diameter, 18 cm upper inside diameter, 20 cm height, and 2 cm thickness) having microelement soil and silt in a 2:1 ratio. Before sowing, seeds were surface sterilized with 0.1% HgCl_2_ solution. Seeds were then rinsed, dried, and subjected to thermopriming by keeping seeds in water at low temperature (4 °C) and high temperature (50 °C) for 1 h. 20 leaves of 10 plants were used for the average sample per treatment. Pots were arranged at 5 cm apart into the control group and heavy metal stress treatment group. After germination, 15 days plants were subjected to foliar spray with α-tocopherol (150 mg/L) solution whereas 10 mL HgCl_2_ solution at different concentrations viz 4 ppm and 8 ppm were directly introduced to pots for imposing heavy metal stress. For physiological and biochemical analyses, true leaf samples were taken during the vegetative stage after 60 days of sowing. For each treatment, three replicates were taken via the method of Ali et al. [43]. Standard procedures for the pot experiments were practiced and during the experiment, no insect or disease issues were noticed. The plant was watered at 25% humidity in pots as needed. Agronomical studies included root, stem, and leaf while physiological parameters were evaluated for leaves that were kept and preserved in a refrigerator at 4 °C for the analysis of osmoprotectants, biochemical and antioxidant enzymes.

### Agronomic characteristics

#### Germination Index (GI)

The germination Index has been computed by the formula described by [44].

$${\text{GI}}=\left(10{\text{Xn}}1\right)+\left(9{\text{xn}}2\right)+\dots +(1{\text{xn}}10)$$

Hence n1, n2... n10 represented the number of seeds germinated on day first, second till the last day 10th whereas; 10, 9... and 1 are values given to the number of sprouted seeds on day first, second, and the until day the last 10th.

#### Coefficient of Velocity of Germination (CVG)

The coefficient of the velocity of germination indicates the speed of germination. The less time it takes to germinate, the higher the CVG value. For such determination, the formula proposed by [54–56] has been followed.

$$\mathrm{CVG N}1 +\mathrm{ N}2 + \cdot \cdot \cdot +\mathrm{ Nx}/100 \times \mathrm{ N}1{\text{T}}1 + \cdot \cdot \cdot +\mathrm{ NxTx}$$

where “N” is the number of germinants per day and “T” is the time counted in days from sowing corresponding to seed geminated N.

#### Mean Emergence Time (MET)

Mean emergence time has been determined by the proposed formula of Javed et al. [57].

$${\text{MET}}=\frac{\mathrm{\Sigma Dn}}{\sum {\text{n}}}$$

where D denotes the number of days since the start of emergence and n denotes the number of seeds that had emerged on day D.

#### Emergence Index (EI)

The emergence index has been determined by the proposed formula of Javed et al. [57].

$${\text{EI}}=\mathrm{No\,of\,emerged\,seeds\,}/\mathrm{\,Day\,of\,first\,the\,count}+..+\mathrm{\,No\,of\,emerged\,seeds}/\mathrm{\,Day\,of\,the\,final\,count}$$

#### Final Germination Percentage (FGP)

The final germination percentage has been determined by the proposed formula of Hakim et al. [58].

$${\text{FPG}}=\mathrm{The\,total\,seeds\,germinated\,at\,the\,end\,of\,the\,trial}/\mathrm{Number\,of\,initial\,seeds\,used\,x}100$$

#### Timson Germination Index (TGI)

Timson germination index has been determined by the proposed formula of Al-Ansari et al. [59].

$${\text{TGI}}=\frac{\sum {\text{G}}}{{\text{T}}}$$

where G is the percentage of seed germinated per day, and T is the germination period.

#### Mean Germination Time (MGT)

The mean germination time was computed by the method of Saeed et al. [54]. Mean germination time indicates seed germination rate. The smaller the meantime of germination,the greater the rate of germination.

$${\text{MGT}}=\frac{\mathrm{\Sigma fx}}{\mathrm{\Sigma f}}$$

where "f" refers to the amount of germinated seeds on day x.

#### Leaf Area Index (LAI) and Leaf Area Ratio (LAR)

The following equation specified the leaf area index and area ratio suggested by Shah et al. [60].

$${\text{LAI}}=\mathrm{Leaf\,area\,}({\text{cm}}2)/\mathrm{\,Land\,area\,}({\text{cm}}2)$$

$${\text{LAR}}=\mathrm{Leaf\,area\,}({\text{cm}}2)/\mathrm{Final\,plant\,dry\,weight}$$

The leaf area was measured by taking the length and width of a leaf and using weighted regression equations for each species to get the leaf area.

#### Root-Shoot Ratio (RSR)

The root-shoot ratio has been determined by the proposed formula of Chuyong and Acidri, [61].

$${\text{RSR}}=\mathrm{Root\,dry\,mass}/\mathrm{Shoot\,dry\,mass}$$

#### Seedling Vigor Index (SVI)

SVI has been calculated using themethod and calculation described by Hatami, [62]; Ullah et al. [63] expressed by means of number and standard deviation.

$${\text{SVI}}=\mathrm{Root\,length}+\mathrm{Shoot\,length\,}/\mathrm{Germination\,Percentage}$$

#### Percent Moisture Content (PMC)

Percent moisture content has been determined by the proposed formula by Ali et al. [43].

$$\mathrm{\%MC}=\mathrm{Wet\,weight\,of\,the\,sample\,}-\mathrm{\,Dry\,weight\,of\,sample}/\mathrm{Dry\,weight\,of\,the\,sample}$$

#### Relative Growth Rate (RGR)

The relative growth rate has been determined by the proposed formula of Shah et al. [60].

$${\text{RGR}}=(\mathrm{loge W}2 -\mathrm{ loge W}1)/{\text{t}}2 -\mathrm{ t}1$$

where, W1 = Weight of dry matter at time t1,W2 = Weight of dry matter at time t2, t2-t1 = the interval in days, loge = Natural logarithms (Logarithms to the base of 2.3026).

#### Absolute Growth Rate (AGR)

The absolute growth rate has been determined by the proposed formula of Shah et al. [64].

$${\text{AGR}}={\text{H}}2-{\text{H}}1/{\text{t}}2-\mathrm{ t}1$$

where H1,and H2 refer to the plant height at the time t1 and t2, respectively.

#### Net Assimilation Rate (NAR)

The net assimilation rate has been determined by the proposed formula of Shah et al. [64].

$${\text{NAR}}={\text{W}}2-{\text{W}}1/\mathrm{ t}2 -\mathrm{ t}1\mathrm{ X }(\mathrm{loge A}2 -\mathrm{ loge A}1)/{\text{A}}2-{\text{A}}1$$

where, A1 and A2 are the leaf areas and W1 and W2 are total dry matter,recorded at times t1 and t2.

#### Crop Growth Rate (CGR)

The crop growth rate has been determined by the proposed formula of Ahmadi et al. [65].

$$\mathrm{Crop growth rate}={\text{W}}2-{\text{W}}1/\mathrm{ t}2 -{\text{t}}1$$

### Physiological and biochemical attributes

#### Determination of total chlorophyll content of leaves (TCC)

The chlorophyll content of fresh leaves is determined by the standard method of Ma et al. [28]. 0.5 g foliar material crushed in 80% acetone. The suspension was centrifuged for 5 min at 2000 rpm. OD was recorded at 663 nm (chlorophyll b), 645 nm (chlorophyll a), and 470 nm (carotenoid).

#### Determination of soluble protein content of leaves (SPC)

Protein content was studied using the recommended method of Kim et al. [66] 0.2 g fresh foliar material was ground in phosphate buffer (pH 7.5). 0.1 mL with 3.0 mL of reagent having CuSO_4_.5H_2_O (0.125 g), (25 mL) and Na–K tartrate (1.5 g), (150 mL) and (3 g) sodium carbonate (Na_2_CO_3_) NaOH (0.4 g), (100 mL) was added. Folin phenol (0.1 mL) reagent has been added after shaking for 10 min. The absorbance for each sample was observed at 650 nm after 30 min incubation.

#### Determination of total proline content of leaves (TPC)

Zhang and Huang’s, [67], method was used for proline estimation in fresh foliar material. In 10 mL of 3% aqueous sulphosalicylic acid, 0.5 g of foliar material was crushed and filtered and 2 mL of filtrate was taken added with 2.0 mL of acid ninhydrin, 2 mL of glacial acetic acid in a test tube and warm it for 1 h at 100 °C in water. The mixture was extracted with 4 mL toluene, and OD was read at 520 nm.

#### Determination of soluble sugar content (SSC)

Sugar content measurements of fresh leaves are obtained using the Marcinska et al. [68] method. With 10 mL of distilled water, fresh foliar materials (0.5 gm) are homogenized and centrifuged for 5 min at 3000 rpm. Adding 1 mL of 30 percent (w/v) phenol to 0.1 mL of supernatant and adding 5 mL of concentrated sulphuric acid after room temperature incubation. The sample was incubated for 4 h and absorbance was recorded at 420 nm.

#### Determination of peroxidase activity (POD)

Ma et al. [28] method was used for POD estimation. Fresh foliar material (0.5 g) was homogenized with 2 mL solution including 0.2 mL phosphate buffer solution (pH 7.0), 12.5 g PVP and 4.6 g (EDTA) in 125 mL distilled water and centrifuged for 20 min. The reaction mixture (3 mL) contained 1.3 mL MES buffer, 0.1 mL phenyl diamine, a drop of 0.3% H_2_O_2_ and 0.1 mL supernatant. Absorbance was registered at 485 nm for 3 min.

#### Determination of superoxide dismutase activity (SOD)

SOD content wasmeasured in fresh foliar material through the standard method of Ma et al. [46]. The reaction mixture (3 mL) contained 0.72 mL methionine, 0.72 mL NBT, EDTA, 0.1 mL supernatant, and 0.72 mL riboflavin followed by a 30 min incubation period in the dark and then in light, and readings were recorded at 560 nm.

#### Determination of malondialdehyde activity (MDA)

Malondialdehyde activity was calculated by the method of Zhang and Huang, [67]. 1 g fresh foliar material was groundwith 1 mL, 0.1% (w/v) Trichloroacetic acid (TCA) and centrifugated for 10 min. 4 mL of 20% Trichloroacetic acid (TCA) containing 0.5% thiobarbituric acid (TBA) has been added to the supernatant. The mixture was boiled for 15 min at 95 °C and cooled on ice. Each sample absorbance was recorded at 532 nm.

#### Determination of catalase activity (CAT)

The activity of catalase was estimated using the method of Ma et al. [28]. Fresh foliar material (0.5 g) was ground and homogenized with buffer in a mortar. 0.2 mL of enzyme extract, 0.4 mL of 30% H_2_O_2_, and 0.4 mL of 100 mM potassium phosphate buffer (pH 7.0) made up the reaction mixture (1 mL). The decrease in absorbance at 240 nm was used to calculate how much H_2_O_2_ was decomposed over a 3-min period.

#### Determination of ascorbate peroxidase (APX)

The activity of ascorbate peroxidase was estimated by the method of Ma et al. [46]. Fresh leaves of 0.5 g were groundand homogenized with 5.0 mL of phosphate buffer. 3.0 mL of the reaction mixture consisting of 1.5 mL phosphate, ascorbate 300 μL, and enzyme extract 600 μL and H_2_O_2_ was taken and absorbance decreases at 290 nm has been recorded.

### Statistical analysis

Statistics 10 and SPSS Statistics 25 were used for statistical analyses. One-way variance analysis (ANOVA) was conducted for variations between three or more means. For Mean separation, Tukey’s multiple comparison test (Tukey's HSD), was conducted using Statistics 10. Analysis of correlation was used to test the positive and negative dependency of two variables. Mean and standard deviation was calculated for agronomic characteristic to test the difference.

## Results and discussion

### Determination of agronomic characteristics

Results of agronomic characteristics mentioned in Table 1 and 2 estimated that the minimum values of EI, CVG, SVI, CGR, NAR,and TGI recorded under 4 ppm concentration of HgCl_2_ indicating the high toxicity of mercury to growth of plants whereas; under 8 ppm results of the mentioned parameters were non-significant with 4 ppm indicated adverse effect of induced mercuric chloride stress forecasting the future yield loss of the crop as a consequence of the soil pollution with heavy metals ahead in the world. The highest was LAI reported at low concentration (Hg 4 ppm) with α-T spray and 4℃ temperature; which is the direct indication of the growth responses in the selected cultivar under induced heavy metal stress, reflecting the second domain of the present study and proposes the idea of using growth regulators to deal with the future situation of soil pollution with heavy metals. Nonetheless, the maximum range for all agronomic characters has been reported after the foliar application of α-T as a growth regulator is an initiative towards the development of heavy metal resistant crops and phytoremediators will be considered the best option to reduce the negative impact of heavy metal pollution in the world.

**Table 1:** Mitigation effect of thermopriming and α-tocopherol on the absolute growth rate, leaf area index, root shoot ratio, percent moisture content, emergence index, coefficient of the velocity of germination, final germination percentage,and mean germination time of *B. napus* under mercuric chloride stress

Treatment	Germination index (GI)	Coefficient of velocity of germination (CVG)	Mean Emergence time (MET)	Emergence index (EI)	Final germination percentage (FGP)	Timson germination index (TGI)	Mean germination time (MGT)	Leaf area index (LAI)
Control	763 ± 126^b^	1115 ± 316^bc^	5.67 ± 0.16^abc^	35.58 ± 6.38^bc^	84.00 ± 8.6^abc^	18.53 ± 1.61^a^	5.85 ± 0.31^a^	2.38 ± 0.4^a^
Hg4ppm	545 ± 58^c^	606 ± 163^d^	5.78 ± 0.26^ab^	24.34 ± 2.40^c^	65.33 ± 11.4^de^	10.13 ± 1.27^e^	5.78 ± 0.26^ab^	2.28 ± 0.5^ab^
Hg8ppm	568 ± 69^bc^	613 ± 144^d^	5.57 ± 0.09^abc^	25.40 ± 3.03^bc^	62.67 ± 7.5^e^	13.90 ± 2.09^bc^	5.67 ± 0.09^abc^	2.13 ± 0.2^ab^
Hg4ppm + 4 °C	591 ± 128^bc^	718 ± 193^ cd^	5.85 ± 0.31^a^	26.52 ± 7.73^bc^	70.67 ± 8.2^cde^	11.03 ± 1.75^cde^	5.67 ± 0.16^abc^	1.93 ± 0.1^ab^
Hg4ppm + 4 °C + tocopherol (150 m/L)	728 ± 118^bc^	933 ± 202^ cd^	5.55 ± 0.21^abcd^	34.31 ± 6.91^bc^	76.00 ± 5.6^bcde^	12.93 ± 1.65^cde^	5.55 ± 0.21^abcd^	2.39 ± 0.7^a^
Hg4ppm + 50 °C	1042 ± 122^a^	1900 ± 265^a^	5.52 ± 0.19^abcd^	48.83 ± 6.97^a^	98.67 ± 1.8^a^	10.33 ± 1.22^de^	5.52 ± 0.19^abcd^	2.07 ± 0.9^ab^
Hg4ppm + 50 °C + tocopherol(150 mg/L)	1080 ± 67^a^	1888 ± 229^a^	5.36 ± 0.01^ cd^	51.52 ± 2.99^a^	96.00 ± 3.2^a^	18.73 ± 1.14^a^	5.36 ± 0.01^dc^	1.98 ± 0.5^ab^
Hg8ppm + 4c	619 ± 40^bc^	723 ± 117^ cd^	5.66 ± 0.07^abcd^	28.70 ± 1.16^bc^	69.33 ± 6.8^cde^	11.27 ± 0.87^cde^	5.66 ± 0.07^abcd^	2.04 ± 0.1^ab^
Hg8ppm + 4 °C + tocopherol(150 mg/L)	761 ± 72^b^	1021 ± 192^ cd^	5.52 ± 0.08^abcd^	36.00 ± 3.48^b^	80.00 ± 8.6^bcd^	13.53 ± 1.29^ cd^	5.52 ± 0.08^abcd^	1.95 ± 0.6^ab^
Hg8ppm + 50 °C	986 ± 109^a^	1532 ± 274^ab^	5.31 ± 0.10^bcd^	47.56 ± 5.57^a^	84.00 ± 6.5^abc^	16.93 ± 1.68^ab^	5.31 ± 0.10^ cd^	2.03 ± 0.2^ab^
Hg8ppm + 50 °C + tocopherol150mg/L)	1006 ± 137^a^	1695 ± 362^a^	5.43 ± 0.13^ cd^	47.96 ± 7.76^a^	90.67 ± 6.8^ab^	17.60 ± 2.06^a^	5.43 ± 0.13^bcd^	1.22 ± 0.7^b^

**Table 2:** Mitigation effect of thermopriming and α-tocopherol on leaf area ratio, seed vigor index, crop growth rate, net assimilation rate, final emergence percentage relative growth rate timson germination index,and germination index of *B. napus* under mercuric chloride stress

Treatment	Leaf area ratio (LAR)	Root shoot ratio (RSR)	Seed vigor index (SVI)	Percent moisture content (PMC)	Relative growth rate (RGR)	Absolute growth rate (AGR)	Net assimilation rate (NAR)	Crop growth rate (CGR)
Control	0.037 ± 0.022^abc^	0.29 ± 0.03^ab^	1541 ± 286^cde^	23.23 ± 0.7^a^	0.037 ± 0.005^a^	0.27 ± 0.09^a^	0.16 ± 0.009^b^	0.28 ± 0.034^a^
Hg4ppm	0.030 ± 0.190^a^	0.24 ± 0.12^ab^	1072 ± 686^e^	16.83 ± 0.7^ab^	0.030 ± 0.014^a^	0.18 ± 0.06^ab^	0.14 ± 0.040^b^	0.26 ± 0.101^a^
Hg8ppm	0.032 ± 0.023^ab^	0.23 ± 0.11^a^	1493 ± 103^cde^	19.68 ± 2.4^ab^	0.034 ± 0.012^a^	0.14 ± 0.06^b^	0.15 ± 0.034^b^	0.25 ± 0.033^a^
Hg4ppm + 4 °C	0.034 ± 0.002^abc^	0.19 ± 0.04^ab^	1621 ± 399^cde^	14.97 ± 4.7^ab^	0.034 ± 0.020^a^	0.13 ± 0.04^b^	0.25 ± 0.135^b^	0.43 ± 0.260^a^
Hg4ppm + 4 °C + tocopherol(150 m/L)	0.025 ± 0.026^abc^	0.16 ± 0.07^ab^	2312 ± 717^abc^	15.86 ± 1.4^ab^	0.025 ± 0.003^a^	0.13 ± 0.01^b^	0.18 ± 0.030^b^	0.39 ± 0.192^a^
Hg4ppm + 50 °C	0.034 ± 0.071^abc^	0.24 ± 0.09^ab^	1169 ± 418^de^	17.63 ± 1.7^ab^	0.034 ± 0.011^a^	0.15 ± 0.04^b^	0.16 ± 0.017^b^	0.29 ± 0.137^a^
Hg4ppm + 50 °C + tocopherol(150 mg/L)	0.022 ± 0.025^bc^	0.15 ± 0.01^ab^	2131 ± 760^abcd^	12.87 ± 7.2^ab^	0.022 ± 0.003^a^	0.18 ± 0.01^ab^	0.25 ± 0.084^b^	0.40 ± 0.017^a^
Hg8ppm + 4 °C	0.034 ± 0.013^abc^	0.18 ± 0.03^ab^	1185 ± 276^de^	14.70 ± 0.4^ab^	0.034 ± 0.011^a^	0.14 ± 0.02^b^	0.23 ± 0.061^b^	0.41 ± 0.113^a^
Hg8ppm + 4 °C + tocopherol(150 mg/L)	0.034 ± 0.055^abc^	0.13 ± 0.01^ab^	2761 ± 697^ab^	16.42 ± 6.2^ab^	0.034 ± 0.008^a^	0.16 ± 0.03^b^	0.28 ± 0.094^b^	0.42 ± 0.020^a^
Hg8ppm + 50 °C	0.037 ± 0.006^abc^	0.23 ± 0.03^ab^	2028 ± 266^bcde^	14.98 ± 0.8^ab^	0.037 ± 0.004^a^	0.16 ± 0.01^b^	0.28 ± 0.039^b^	0.49 ± 0.125^a^
Hg8ppm + 50 °C + tocopherol(150 mg/L)	0.032 ± 0.029^c^	0.11 ± 0.03^b^	3122 ± 194^a^	8.02 ± 1.61^b^	0.032 ± 0.002^a^	0.13 ± 0.03^b^	0.62 ± 0.297^a^	0.49 ± 0.083^a^

According to the results of variance analysis (Table 3), the maximum effect of treatment was recorded on CVG (1900) for 4 ppm and 50 °C thermopriming at a significant level of *p* < 0.05. EI maximum for 4 ppm, 50 °C thermopriming, and α-T (150 m/L) at a high significant level (*p* < 0.01). FGP was recorded in the treatment of 4 ppm 50 °C at a significant level of *p* < 0.05, whereas; NAR maximum effect for 8 ppm mercuric chloride, 50 °C thermopriming, and α-T (150 m/L) at a significant level (*p* < 0.05). SVI showed significance at *p* < 0.05 for 8 ppm, 50 °C thermopriming,and α-T (150 m/L). The TGI maximum effect for treatment was recorded for mercuric chloride (8 ppm), 50 °C thermopriming, and α-T (150 mg/L) at a significant level of *p* < 0.01.

**Table 3:** Analysis of variance of the measured agronomic traits under mercuric chloride stress

Trait	Source of variation	SS	*Df*	MS	*F*	*p*
AGR	Treatment	0.0513	10	0.00513	1.77	0.1264
	Error	0.06367	22	0.00289	–	–
CGR	Treatment	0.21567	10	0.02157	0.92	0.534
	Error	0.51633	22	0.02347	–	–
CVG	Treatment	7704592	10	770459	9.31	0
	Error	1821086	22	82777	–	–
EI	Treatment	3198.47	10	319.847	7.21	0.0001
	Error	975.87	22	44.358	–	–
FGP	Treatment	4446.06	10	444.606	5.56	0.0004
	Error	1760	22	80	–	–
GI	Treatment	1249800	10	124980	8.17	0
	Error	336479	22	15,294	–	–
LAI	Treatment	3.0254	10	0.30254	0.66	0.7446
	Error	10.0216	22	0.45553	–	–
LAR	Treatment	0.08587	10	0.00859	1.32	0.28
	Error	0.14307	22	0.0065	–	–
MET	Treatment	0.85902	10	0.0859	2.01	0.0835
	Error	0.94233	22	0.04283	–	–
MGT	Treatment	0.85902	10	0.0859	2.01	0.0835
	Error	0.94233	22	0.04283	–	–
NAR	Treatment	0.55893	10	0.05589	3.11	0.0127
	Error	0.39557	22	0.01798	–	–
PMC	Treatment	439.74	10	43.9738	0.58	0.8162
	Error	1680.54	22	76.3883		
RGR	Treatment	0.00081	10	8.094	0.53	0.8528
	Error	0.00338	22	1.536		
RSR	Treatment	0.08147	10	0.00815	1.2	0.3407
	Error	0.14883	22	0.00677		
SVI	Treatment	1.389	10	1389326	3.82	0.0042
	Error	7994145	22	363370		
TGI	Treatment	331.422	10	33.1422	9.11	0
	Error	80.08	22	3.64		

^*^AGR Absolute growth rate, *LAI* Leaf area index, *RSR* Root shoot ratio, *PMC* Percent moisture content, *EI* Emergence index, *CVG* Coefficient of velocity of germination, *FGP* Final germination percentage, *MGT* Mean germination time, *LAR* Leaf area ratio, *SVI* Seed vigor index, *CGR* Crop growth rate, *NAR* Net assimilation rate, *RGR* Relative growth rate, *TGI* Timson germination index, *GI* Germination index, *MET* Mean emergence time

Aggregation of heavy metals in *B. napus* has been suggested for use to clean up soil from heavy metals. Germination assay is a basic means of limiting the toxic problem of heavy metals. As we found that the growth of *B. napus* was adversely impacted by higher amounts of Hg (8 ppm) relative to the corresponding controls. Previously, the tolerance of seedling growth was well explained in cotton by Iqbal et al. [69] and barley by Qadir et al. [70]. Heavy metal stress affects several plant species [71]; some plant species can bear heavy metal stress. For example, this investigation examined the effects of metal stress on growth, physiological and biochemical performance, and the mitigation of metal-induced damages in *B. napus* using alpha-tocopherol which eventually impacts crop health. Results of agronomic characteristics (AGR, PMC, and MGT) recorded minimum under high mercuric chloride concentration (8 ppm) treatment indicating the adverse effect of induced mercuric chloride stress forecasting the future yield loss of the crop as a consequence of the soil pollution with heavy metals ahead in the world. However, high heavy metal stress of 8 ppm concentration affected plant growth negatively both in the existence as well as an absence of α-T and temperature. An rise in mercury content up to 7 mm resulted in the greatest percentage of seed germination reduction (42%), seedling length (70%), root length (66%), and seedling dry weight (47%) concerning control by Daud et al. [72].

### Physiological and biochemical attributes analysis

**Fig. 1:**
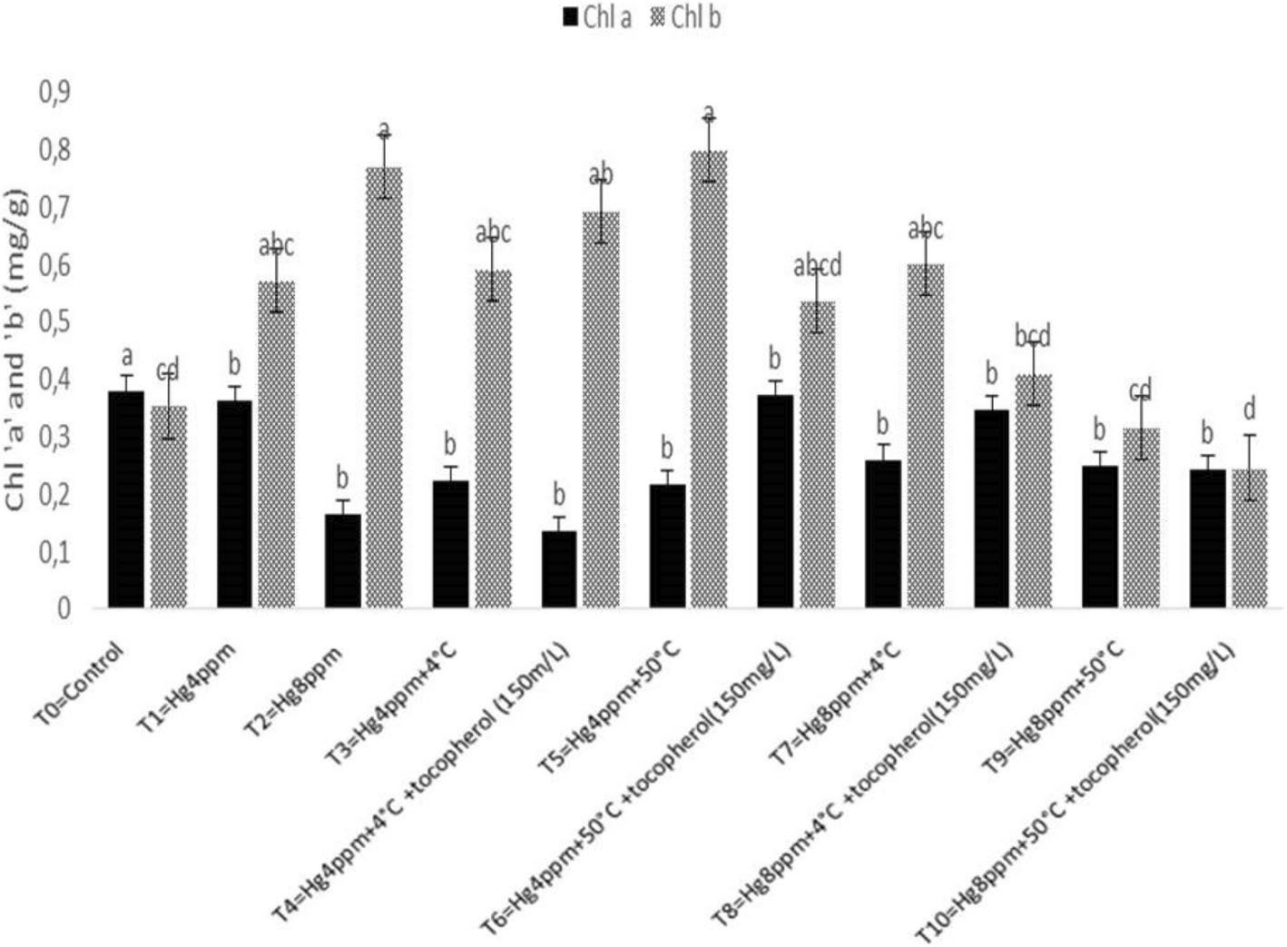
Mitigation effect of α-tocopherol on chlorophyll a (Chl a) and chlorophyll b (Chl b) of *B. napus* under mercuric chloride and temperature stress

**Fig. 2:**
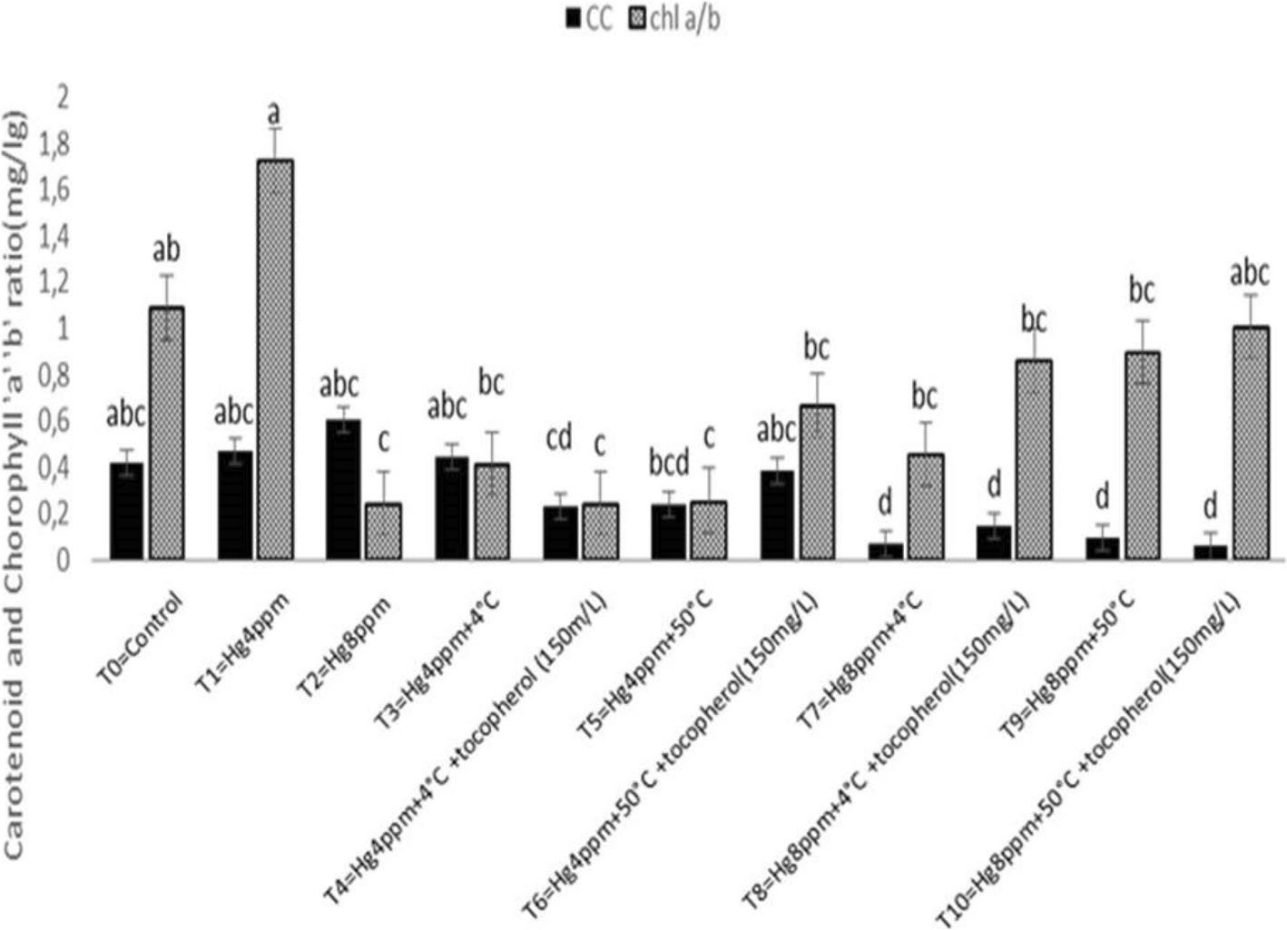
Mitigation effect of α-tocopherol on chlorophyll a/b (Chl a/b) and Carotenoid (CC) of *B. napus* under mercuric chloride and temperature stress

**Fig. 3:**
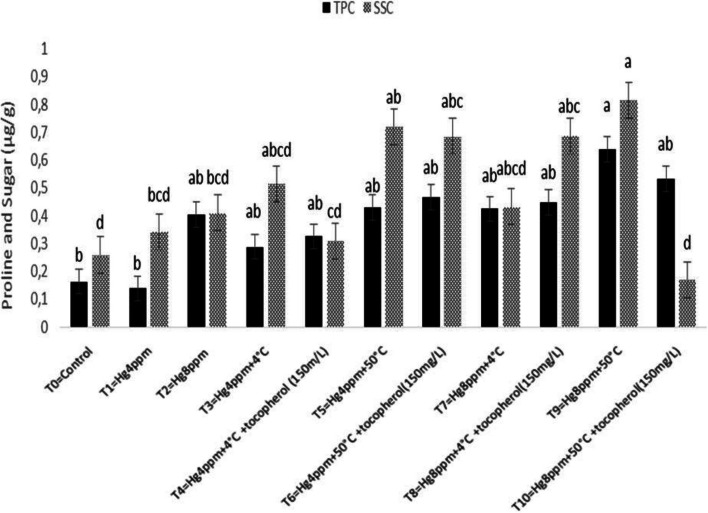
Mitigation effect of α-tocopherol on total proline content (TPC) and soluble sugar content (SSC) of *B. napus* under mercuric chloride and temperature stress

Estimation of photosynthetic pigment in leaves is a key technique for correctly monitoring plant stress levels, which vary between species and are mostly reliant on soil water content, CO_2_ in the air, sunshine intensity, and temperature. The results shown in Fig. 1 demonstrated that Chl "a" rise under generated mercuric chloride stress of 4 ppm concentration and thermopriming therapy (50 °C) at T5 = Hg4ppm + 50 °C, however Chl "b" increased non-significantly under the same conditions. The maximum Chl a/b ratio was determined at 4 ppm treatment, whereas carotenoid content (CC) increased under high-stress conditions, indicating the lowest growth response in selected varieties, reflecting susceptibility at the highest level of induced mercuric chloride stress under natural conditions as well as with the application of growth regulators (Fig. 2). Similarly, TPC increased significantly at *p* < 0.05, and SSC increased at the maximum mercuric chloride stress of 8 ppm, demonstrating that sugar and proline act as osmoprotectants fast and effectively when plants are exposed to harsh stress conditions, as shown in Fig. 3. All plants have detoxification processes for reactive oxygen species, which can be classified as non-enzymatic or enzymatic.

**Fig. 4:**
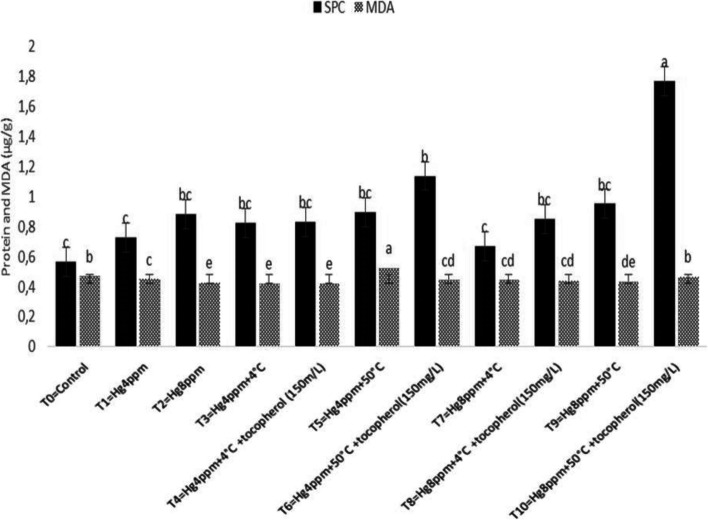
Mitigation effect of α-tocopherol on soluble protein content (SPC) and malondialdehyde (MDA) of *B. napus* under mercuric chloride and temperature stress

Under metal stress conditions, there is a great reduction in the activity of antioxidant enzymes with a net increase in free amino acids due to the degradation of protein and depression in its synthesis in plants. In Fig. 4 results indicated that SPC has a maximum value at *p* < 0.05 significant for 8 ppm stress under 50 °C thermopriming with α-T spray while MDA at 4 ppm mercury concentration under the same priming condition reflecting that the lowest metal stress was quenched through priming treatment indicating no net decrease in protein content showed an association with antioxidant enzymes that possibly increased.

**Fig. 5:**
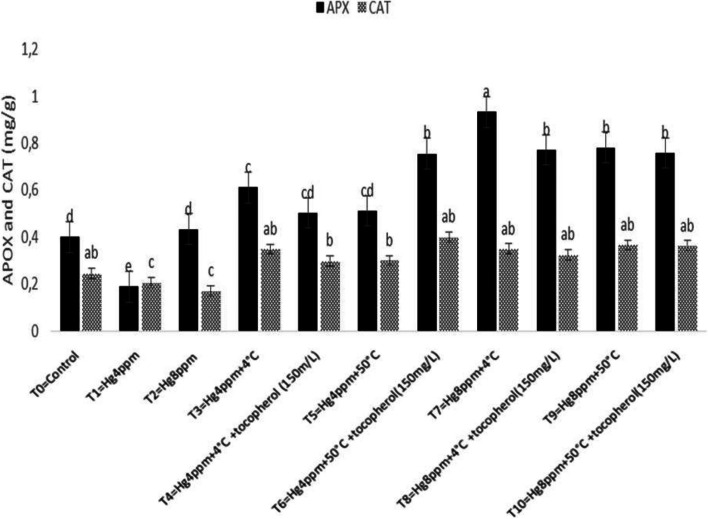
Mitigation effect of α-tocopherol on ascorbate peroxidase (APX) and catalase (CAT) of *B. napus* under mercuric chloride and temperature stress

**Fig. 6:**
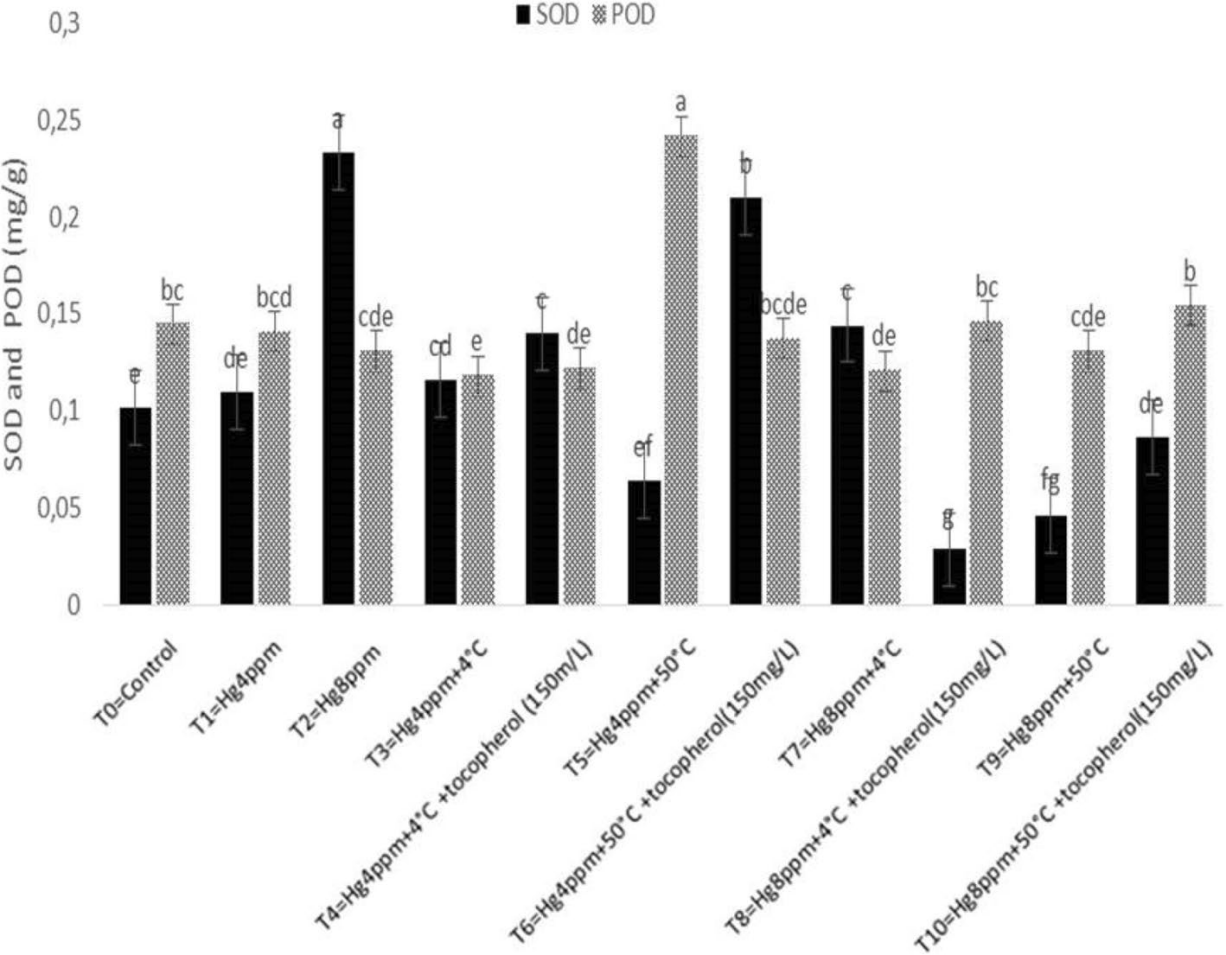
Mitigation effect of α-tocopherol on superoxide dismutase (SOD) and peroxidase (POD) of *B. napus* under mercuric chloride and temperature stress

Antioxidant mechanisms exist in all plant species by protecting themselves from the harsh environmental condition that produces ROS species through their antioxidant system either non-enzymatic or enzymatic [73, 74]. Results in Fig. 5 revealed the maximum value for CAT and APX by thermopriming at chilling temperature (4 °C) under 4 pmm and 8 ppm concentrations significantly at *p* < 0.05. Similarly, the lowermost of CAT and AP*X* activities have been reported in treatment with 4 ppm and 8 ppm mercury representing that pre-sowing temperature initiated some metabolic processes in the plant that reduce the negative impacts caused by low metal stress and can be resistant by this specie respectively. Nonetheless, results in Fig. 6 described that SOD has a maximum value for 8 ppm mercury concentration, and POD under 4 ppm stress condition via 50 °C thermopriming treatment. Results indicated an increase in SOD and POD enzyme activity that might represent a high level of H_2_O_2_ synthesis and consumption in the proposed specie under stress conditions.

The ability of metal stress on the concentration of chlorophyll, carotenoid content,and formation of reactive oxygen species (ROS) in the thylakoid’s membrane adversely affects plant growth response [75, 76]. The findings of the present study demonstrated a minimum of Chl “b” and CC reported in the treatment (Hg 8 ppm + 50 °C + α-T) suggested that heavy metal stress resulted in decrease in CC due to the poor absorption of mineral ions necessary for maintaining optimum osmotic potential in plants that have been disturbed by mercury cations. Rendering to the evaluation, Ali et al. [43] analyze the agronomical, physiological, and biochemical mechanisms accompanying the acquisition of *Vigna radiata* L. variety ‘Ramzan’ using seed osmo- and thermopriming in the presence of PEG-4000 and 4 °C. Similar techniques in the experiment were also described by [61]. They reported the effect of thermopriming on physiological, biochemical, and antioxidant activities in staple food crop (*Triticum aestivum* L.). Our results are also in agreement with the work of Alsherif et al. [77]. They also stated the antioxidant defense molecules including alpha and beta tocopherols *Sesuvium portulacastrum* L.) against heavy metal toxicity. Physiological attributes like chlorophyll, carotenoid, proline, total soluble sugars, and antioxidant enzymes were also studied by Zhang et al. [78] in duckweed and supported our results, only chlorophyll content (mg g^−1^) studied by Skudra and Ruza [79], in winter wheat, while Zhang and Huang, [67], studied chlorophyll proline and soluble sugar in *Arabidopsis* seedling, chlorophyll a/b at seedling stage and total chlorophyll content at anthesis stage also done by Rehman et al. [80] in *T. aestivum*, similarly the work of Arjenaki et al. [81] also supported our results and determined chlorophyll content, relative water content, and minerals content in *T. aestivum* under drought stress. A bit change in an increased level of total soluble sugar (SSC) and total proline content (TPC) under metal stress (8 ppm) was significant in represented species evaluated that plant initiates osmoprotective mechanism for the production of osmolytes e.g. sugar and proline etc. against severe damages caused by oxidation of biomolecules under stress situation. Oxidative stress created in stress blocks the growth and development by disturbing the cell cycle (cell division), and physiology, hence defense from such oxidative stress is serious for the germination of the seed [82, 83]. Increased level of SOD and POD under high mercury concentration (8 ppm) whereas the maximum value of CAT at treatment (Hg 4 ppm + 50 °C + α-T) and AP*X* at 8 ppm and 4 °C thermopriming suggesting the large formation of oxidants and its consumption under metal stress.

### Analysis of variance of the measured traits

According to the results of variance analysis (Table 4), the maximum effect of treatment was recorded on MDA (4.22) and showed no significance followed by SPC, treatment (1.265) at a high significant level (*p* < 0.01). TCC was recorded in treatment (0.88) at the significant level of *p* < 0.05, whereas; carotenoid content (CC) and TPC (0.745, 0.526) under mercuric chloride stress at a significant level (*p* < 0.01).

**Table 4:** Analysis of variance of the measured traits under mercuric chloride stress

Trait	Source of variation	SS	*Df*	MS	*F*	*p*
Chl "a"	Treatment	0.230	1	0.23	2.163	0.151
	Error	3.293	31	0.106	–	–
Chl "b"	Treatment	0.210	1	0.21	4.125	0.051
	Error	1.581	31	0.051	–	–
Chl a/b ratio	Treatment	0.050	1	0.05	0.136	0.715
	Error	11.417	31	0.368	–	–
TCC	Treatment	0.880	1	0.88	6.034	0.02
	Error	4.521	31	0.146	–	–
CC	Treatment	0.745	1	0.745	32.826	0
	Error	0.704	31	0.023	–	–
SSC	Treatment	0.169	1	0.169	2.218	0.147
	Error	2.358	31	0.076	–	–
SPC	Treatment	1.265	1	1.265	12.781	0.001
	Error	3.069	31	0.099	–	–
TPC	Treatment	0.526	1	0.526	11.099	0.002
	Error	1.47	31	0.047	–	–
POD	Treatment	0.034	1	0.034	3.58	0.068
	Error	0.298	31	0.01	–	–
SOD	Treatment	0.087	1	0.087	3.583	0.068
	Error	0.753	31	0.024	–	–
CAT	Treatment	0.049	1	0.049	10.971	0.002
	Error	0.14	31	0.005	–	–
APX	Treatment	1.004	1	1.004	53.864	0
	Error	0.578	31	0.019	–	–
MDA	Treatment	0.22	1	0.22	0.007	0.932
	Error	0.177	31	0.006		

^*^Chl “a” Chlorophyll “a”, *Chl “b”* Chlorophyll “b”, *Chl a/b ratio* Chlorophyll a/b ratio, *TCC* Total chlorophyll content, *CC* Carotenoid content, *SSC* Soluble sugar content, *SPC* Soluble protein content, *TPC* Total proline content, *POD* Peroxidase, *SOD* Superoxide dismutase, *CAT* Catalase, *APX* Ascorbate peroxidase, *MDA* Malondialdehyde

### Principal component analysis of the biological components

**Fig. 7:**
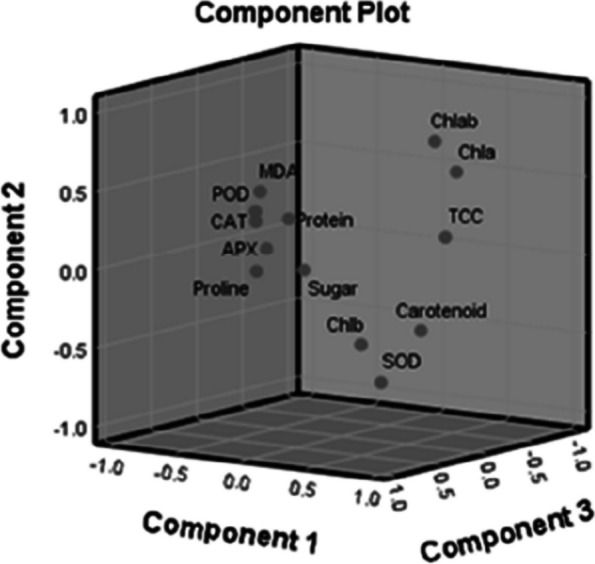
Loading plots of PC1, PC2 and PC3 in rotated space

Table 5 and Fig. 7 represent principal component analysis. The results were based on 13 characters and represented that the first PC1 explained 27.877% of the complete variance, which was significantly correlated with TCC, Chl ‘‘b’’, CC, and SOD particularly associated with growth responses. However, the second PC2 explained 19.938% of the complete variance and correlated with Chl ab ratio, Chl a, SPC, AP*X*, and CAT corresponded to plant growth regulator. Similarly, PC3 accounted for 14.317% of entire variations with important variables being POD, MDA, sugar, and proline, hence particularly related toantioxidant enzymes.

**Table 5:** Eigenvalues, variation explained (%), cumulative variance (%), coefficients of determination of the first three principal components based on the correlation matrix of biological components

Traits	Eigen values	Variance (%)		Components		
		**Individuals**	**Cumulative**	**PC1**	**PC2**	**PC3**
Chl 'a'	3.903	27.877	27.877	0.524	0.728	-0.288
Chl 'b'	2.791	19.938	47.815	0.550	-0.238	0.486
Chl ab	2.004	14.317	62.133	0.220	0.779	-0.463
TCC	1.506	10.760	72.893	0.741	0.451	0.047
CC	1.038	7.414	80.307	0.738	-0.190	0.092
SPC	0.813	5.805	86.112	-0.387	0.007	-0.004
TPC	0.686	4.904	91.015	-0.496	-0.250	0.149
SSC	0.451	3.218	94.234	-0.208	-0.042	0.129
CAT	0.372	2.654	96.888	-0.760	0.026	-0.092
AP*X*	0.211	1.505	98.393	-0.691	-0.224	-0.228
MDA	0.151	1.079	99.471	-0.201	0.494	0.775
SOD	0.043	0.309	99.780	0.548	-0.693	0.058
POD	.031	.220	100.000	-0.139	0.385	0.870

^*^Chl “a” Chlorophyll “a”, *Chl “b”* Chlorophyll “b”, *Chl ab* Chlorophyll a/b ratio, *TCC* Total chlorophyll content, *CC* Carotenoid content, *SPC* Soluble protein content, *TPC* Total proline content, *SSC* Soluble sugar content, *POD* Peroxidase, *APX* Ascorbate peroxidase, *MDA* Malondialdehyde, *SOD* Superoxide dismutase, *CAT* Catalase

### Correlation analysis of physiological parameters

Analysis of correlation Table 6 estimated the positive correlation of chl “a” with Chl “a/b” ratio and TCC and Chl “a” at significant level (*p* = 0.01). Chl b is positively correlated with the ratio of Chl “a/b”, CC, and SOD at a significant level (*p* = 0.05) and Chl a/b ratio is positively correlated with TCC at a significant level (*p* = 0.01). TCC is negatively correlated with TPC, CAT, and AP*X* at a significant level (*p* = 0.05) and CC is negatively correlated with CAT at a significant level (*p* = 0.05) and with APX at a significant level (*p* = 0.01) while positively correlated with SOD at a significant level (*p* = 0.01). TPC is positively correlated with CAT and AP*X* at a significant level (*p* = 0.05), CAT is positively correlated with AP*X* and negative correlated with SOD at a significant level (*p* = 0.01) and MDA is positively correlated with SOD at a significant level (*p* = 0.05). Recent trends in agriculture have validated the use of plant growth regulators, to surge the growth and yield of crop plants [84]. Today mankind has the highest priority in facing climate change problems that influence agricultural growth with water scarcity, so plants expected difficulties to grow and survive worldwide in such a situation. The pollution of heavy metal ions in the atmosphere will adversely impact beneficial soil microbial communities and interrupt the nitrification, denitrification, and decomposition of organic matter. Protecting water and soil from heavy metal contaminants and thereby protecting human health and other habitats around the planet [85]. Mercury (Hg) contamination has gained particular interest due to Hg's high toxicity and frequent occurrence described by Zhang et al. [78]. Between the pollution-producing metals Mercury (Hg) and cadmium (Cd) are considered nonessential with no documented physiological functions. These are greatly toxic to organisms such as plants and animals have a prolonged half-life and are tenacious in a number of ecosystems [49]. Accordingly, Mwamba et al. [86] examined the accumulation of heavy metals in mustard and their effects on plant growth, biomass, and physiological processes in the plant. Lead heavy metal toxicity led to changes in plant growth and antioxidative enzyme levels in the water. Lead is neither a necessary nor a helpful component for plant growth [87]. However, because heavy metals have detrimental effects on plant development and growth, their temporal ccumulation at higher concentrations in waste-amended agricultural soils can be harmful for plant growth [53]. The detrimental effects of heavy metals on plant growth and development provide an explanation for this. Enzymatic reactions like POD, CAT, AP*X*, GR, and SOD indicate both the level of toxicity and the ability of plants to tolerate toxic stress described by Elbaz et al. [88]. Although the outcomes pertaining to biochemical activities are associated with Zhang et al. [78] and Pravisya and Jayaram, [89]. Some study also revealed the agronomic activities, thermo and osmopriming on mung bean under mercuric chloride stress tolerance also reported by Ahmad et al. [49] whose study in line with our findings. Plant photosynthetic pigments are responsible for the crop's growth percentage. Similarly, Javed et al. [90] conducted a study that showed that when AP*X* antioxidants were applied to the seedlings, it helped increase the cell division rate and seedling growth rate under stressful environmental conditions. This suggests that AP*X* antioxidants can help protect against environmental stress and help improve seedling growth [6, 14, 91–93]. Zhang et al. [78] also agree with our results and proved that mercury can induce oxidative stress and can activate an anti-oxidative system in duckweed. Elbaz et al. [88] also support our results and studied the impact on antioxidant enzymes in *Chlamydomonas reinhardtii* under Mercury-induced oxidative stress, while Kim et al. [66] determined the effect of mercury on seed germination and seedling growth of *V. radiata*. Our results are consistent with the findings of Zhai et al. [51], who observed that the activities of CAT, SOD, PPO (polyphenol oxidase), and APX improved in micropropagated plants while POX (peroxidase) and ASO (ascorbate oxidase) decreased. The growth and physiology of *B. napus* plants are negatively impacted by high concentrations of mercuric chloride, which is controlled by α-T spray in test species.

**Table 6:** Correlation analysis of physiological components

	**Chl “a”**	**Chl “b”**	**Chl a/b**	**TCC**	**CC**	**SPC**	**TPC**	**SSC**	**CAT**	**APO*X***	**MDA**	**POD**	**SOD**
Chl “a”	1.0												
Chl “b”	0.017	1.0											
Chl a/b ratio	0.866**	0.405*	1.0										
TCC	0.963**	0.288	0.720**	1.0									
CC	0.239	0.358*	-0.005	0.326	1.0								
SPC	-0.107	-0.32	0.064	-0.189	-0.251	1.0							
TPC	-0.326	-0.164	-0.222	-0.356*	-0.154	0.265	1.0						
SSC	-0.086	0.076	-0.145	-0.062	-0.19	-0.283	0.188	1.0					
CAT	-0.29	-0.29	-0.154	-0.356*	-0.421*	0.233	0.360*	0.203	1.0				
APOX	-0.331	-0.263	-0.2	-0.388*	-0.556**	0.299	0.368*	0.185	0.585**	1.0			
MDA	0.034	0.016	0.035	0.037	-0.123	0.157	0.061	-0.026	0.072	-0.121	1.0		
POD	-0.019	0.158	-0.065	0.025	-0.103	0.175	0.127	0.09	-0.026	-0.16	0.919**	1.0	
SOD	-0.165	0.380*	-0.323	-0.055	0.535**	-0.012	0.003	-0.11	-0.475**	-0.18	-0.325	-0.226	1.0

^*^Chl “a” Chlorophyll “a”, *Chl “b”* Chlorophyll “b”, *Chl a/b ratio* Chlorophyll a/b ratio, *TCC* Total chlorophyll content, *CC* Carotenoid content, *SSC* Soluble sugar content, *SPC* Soluble protein content, *TPC* Total proline content, *CAT* Catalase, *APOX* Ascorbate peroxidase, *MDA* Malondialdehyde, *POD* Peroxidase, *SOD* Superoxide dismutase

^**^Correlation is significant at the 0.01 level (2-tailed). *correlation is significant at the 0.05 level (2-tailed)

## Conclusion

Pakistan is an agricultural and developing country that is facing scarcity of nutritional food supply due to poor irrigation systems and contamination of agricultural land by toxic metal supply from industries that cannot sustain adequate food production for a growing and increasingly affluent population. The goal of the current study is to reduce the harm done to rapeseed (*B. napus* L.) by heavy metal ions (Mercury) using a growth regulator (α-tocopherol 150 mg/L) and thermopriming technique at 4 °C and 50 °C to preserve plant agronomic and physiological features. Results showed that under 4 ppm mercuric chloride stress, the antioxidant system was initiated by high temperature, reducing photosystem damage. Chlorophyll content was enhanced up to the maximum level at 50 °C thermopriming with growth regulator and antioxidant enzymes like catalase, peroxidase, and malondialdehyde. Under high mercuric chloride stress (8 ppm), however, protein, proline, superoxide dismutase, and ascorbate peroxidase increased non-significantly (*p* > 0.05), indicating the tolerance of selected specie by synthesising osmolytes to resist oxidation mechanism. Additionally, foliar application of α-tocopherol, 50 °C thermopriming, and 4 ppm heavy metal stress can easily improve the reduction in % MC with a notable rise in plant vigour and germination energy. Therefore the findings concluded that the exogenous application of α-tocopherol and thermopriming technique, which synthesises high levels of proline and antioxidant activities to maintain seedling growth and development on heavy metal-contaminated land, can ameliorate the inhibitory effect of even lower concentrations of heavy metal stress (4 ppm).

## Data Availability

All data generated or analyzed during this study are included in this published article.

## Abbreviations

GI: Germination index
CVG: Coefficient of velocity of germination
MET: Mean Emergence time
EI: Emergence index
FGP: Final germination percentage
TGI: Timson germination index
MGT: Mean germination time
LAI: Leaf area index
LAR: Leaf area ratio
RSR: Root shoot ratio
SVI: Seed vigor index
PMC: Percent moisture content
RGR: Relative growth rate
AGR: Absolute growth rate
NAR: Net assimilation rate
CGR: Crop growth rate

## References

[1] Lobell DB, Gourdji SM (2012). The influence of climate change on global crop productivity. Plant Physiol.

[2] Haq IU, Ullah S, Amin F, Nafees M, Shah W, Ali B, Iqbal R, Kaplan A, Ali MA, Elshikh MS (2023). Physiological and Germination Responses of Muskmelon (Cucumis melo L.) Seeds to Varying Osmotic Potentials and Cardinal Temperatures via a Hydrothermal Time Model. ACS Omega.

[3] Javed MA, Khan MN, Ali B, Wahab S, Din IU, Razak SA: Positive and Negative Impacts of Biochar on Microbial Diversity. In: *Sustainable Agriculture Reviews 61: Biochar to Improve Crop Production and Decrease Plant Stress under a Changing Climate.* Springer; 2023: 311–330.

[4] Abeed AH, Tammam SA, El-Mahdy M (2022). Hydrogen peroxide pretreatment assisted phytoremediation of sodium dodecyl sulfate by Juncus acutus L. BMC Plant Biol.

[5] Raza MAS, Ibrahim MA, Ditta A, Iqbal R, Aslam MU, Muhammad F, Ali S, Çiğ F, Ali B, Muhammad Ikram R (2023). Exploring the recuperative potential of brassinosteroids and nano-biochar on growth, physiology, and yield of wheat under drought stress. Sci Rep.

[6] Ali B, Hafeez A, Javed MA, Afridi MS, Abbasi HA, Qayyum A, Batool T, Ullah A, Marc RA, Al Jaouni SK (2022). Role of endophytic bacteria in salinity stress amelioration by physiological and molecular mechanisms of defense: A comprehensive review. S Afr J Bot.

[7] Dhir B (2018). Crop productivity in changing climate. Sustain Agric Rev.

[8] Solangi F, Zhu X, Khan S, Rais N, Majeed A, Sabir MA, Iqbal R, Ali S, Hafeez A, Ali B (2023). The global dilemma of soil legacy phosphorus and its improvement strategies under recent changes in agro-ecosystem sustainability. ACS Omega.

[9] Aragão LE, Poulter B, Barlow JB, Anderson LO, Malhi Y, Saatchi S, Phillips OL, Gloor E (2014). Environmental change and the carbon balance of A mazonian forests. Biol Rev.

[10] Change IC: Impacts, adaptation and vulnerability. *Part A: global and sectoral aspects Contribution of working group II to the fifth assessment report of the intergovernmental Panel on Climate Change* 2014, 1132.

[11] Khan MA, Asaf S, Khan AL, Jan R, Kang S-M, Kim K-M, Lee I-J (2020). Thermotolerance effect of plant growth-promoting Bacillus cereus SA1 on soybean during heat stress. BMC Microbiol.

[12] Ayaydin F, Bíró J, Domoki M, Ferenc G, Fehér A (2015). Arabidopsis NAP-related proteins (NRPs) are soluble nuclear proteins immobilized by heat. Acta Physiol Plant.

[13] Shumaila S, Ullah S (2020). Mitigation of salinity-induced damages in Capsicum annum L.(sweet pepper) seedlings using priming techniques: a future perspective of climate change in the region. Commun Soil Sci Plant Anal.

[14] Ali B, Hafeez A, Ahmad S, Javed MA, Afridi MS, Dawoud TM, Almaary KS, Muresan CC, Marc RA, Alkhalifah DHM (2022). Bacillus thuringiensis PM25 ameliorates oxidative damage of salinity stress in maize via regulating growth, leaf pigments, antioxidant defense system, and stress responsive gene expression. Front Plant Sci.

[15] Vijayakumari K, Jisha K, Puthur JT (2016). GABA/BABA priming: a means for enhancing abiotic stress tolerance potential of plants with less energy investments on defence cache. Acta Physiol Plant.

[16] Shumaila, Ullah S, Shah W, Hafeez A, Ali B, Khan S, Ercisli S, Al-Ghamdi AA, Elshikh MS. Biochar and Seed Priming Technique with Gallic Acid: An Approach toward Improving Morpho-Anatomical and Physiological Features of Solanum melongena L. under Induced NaCl and Boron Stresses. ACS Omega. 2023;8(31):28207–28232. 10.1021/acsomega.3c01720 PMC10763624 38173954

[17] Ali Q, Shabaan M, Ashraf S, Kamran M, Zulfiqar U, Ahmad M, Zahir ZA, Sarwar MJ, Iqbal R, Ali B (2023). Comparative efficacy of different salt tolerant rhizobial inoculants in improving growth and productivity of Vigna radiata L. under salt stress. Sci Rep.

[18] Jisha K, Vijayakumari K, Puthur JT (2013). Seed priming for abiotic stress tolerance: an overview. Acta Physiol Plant.

[19] Kumar V, Sah SK, Khare T, Shriram V, Wani SH. Engineering phytohormones for abiotic stress tolerance in crop plants. Plant hormones under challenging environmental factors. 2016:247–266.

[20] Hafeez A, Ali B, Javed MA, Saleem A, Fatima M, Fathi A, Afridi MS, Aydin V, Oral MA, Soudy FA (2023). Plant breeding for harmony between sustainable agriculture, the environment, and global food security: an era of genomics-assisted breeding. Planta.

[21] Farooq TH, Rafay M, Basit H, Shakoor A, Shabbir R, Riaz MU, Ali B, Kumar U, Qureshi KA, Jaremko M (2022). Morpho-physiological growth performance and phytoremediation capabilities of selected xerophyte grass species toward Cr and Pb stress. Front Plant Sci.

[22] Ahmed T, Masood HA, Noman M, Al-Huqail AA, Alghanem SM, Khan MM, Muhammad S, Manzoor N, Rizwan M, Qi X (2023). Biogenic silicon nanoparticles mitigate cadmium (Cd) toxicity in rapeseed (Brassica napus L.) by modulating the cellular oxidative stress metabolism and reducing Cd translocation. J Hazardous Mater.

[23] Tiwari S, Lata C (2018). Heavy metal stress, signaling, and tolerance due to plant-associated microbes: an overview. Front Plant Sci.

[24] Bibi S, Ullah S, Hafeez A, Khan M, Javed M, Ali B, Din I, Bangash S, Wahab S, Wahid N. Exogenous Ca/Mg quotient reduces the inhibitory effects of PEG induced osmotic stress on Avena sativa L. Brazilian J Biol. 2022;84. 10.1590/1519-6984.264642 36169411

[25] Afridi MS, Javed MA, Ali S, De Medeiros FHV, Ali B, Salam A, Marc RA, Alkhalifah DHM, Selim S, Santoyo G (2022). New opportunities in plant microbiome engineering for increasing agricultural sustainability under stressful conditions. Front Plant Sci.

[26] Ma J, Saleem MH, Ali B, Rasheed R, Ashraf MA, Aziz H, Ercisli S, Riaz S, Elsharkawy MM, Hussain I (2022). Impact of foliar application of syringic acid on tomato (Solanum lycopersicum L.) under heavy metal stress-insights into nutrient uptake, redox homeostasis, oxidative stress, and antioxidant defense. Front Plant Sci.

[27] Ma J, Saleem MH, Yasin G, Mumtaz S, Qureshi FF, Ali B, Ercisli S, Alhag SK, Ahmed AE, Vodnar DC (2022). Individual and combinatorial effects of SNP and NaHS on morpho-physio-biochemical attributes and phytoextraction of chromium through Cr-stressed spinach (Spinacia oleracea L.). Front Plant Sci.

[28] Ma J, Ali S, Saleem MH, Mumtaz S, Yasin G, Ali B, Al-Ghamdi AA, Elshikh MS, Vodnar DC, Marc RA (2022). Short-term responses of Spinach (Spinacia oleracea L.) to the individual and combinatorial effects of Nitrogen, Phosphorus and Potassium and silicon in the soil contaminated by boron. Front Plant Sci.

[29] Regier N, Larras F, Bravo AG, Ungureanu V-G, Amouroux D, Cosio C (2013). Mercury bioaccumulation in the aquatic plant Elodea nuttallii in the field and in microcosm: accumulation in shoots from the water might involve copper transporters. Chemosphere.

[30] Gworek B, Dmuchowski W, Baczewska-Dąbrowska AH (2020). Mercury in the terrestrial environment: A review. Environ Sci Eur.

[31] Wang J, Zhao Y, Zhang P, Yang L, Xi G (2018). Adsorption characteristics of a novel ceramsite for heavy metal removal from stormwater runoff. Chin J Chem Eng.

[32] Prajapati R, Kataria S, Jain M (2020). Seed priming for alleviation of heavy metal toxicity in plants: an overview. Plant Science Today.

[33] Alshegaihi RM, Mfarrej MFB, Saleem MH, Parveen A, Ahmad KS, Ali B, Abeed AH, Alshehri D, Alghamdi SA, Alghanem SM (2023). Effective citric acid and EDTA treatments in cadmium stress tolerance in pepper (Capsicum annuum L.) seedlings by regulating specific gene expression. South African J Botany.

[34] Mahmood T, Islam K, Muhammad S (2007). Toxic effects of heavy metals on early growth and tolerance of cereal crops. Pak J Bot.

[35] Amin N-U, Ahmad T (2015). Contamination of soil with heavy metals from industrial effluent and their translocation in green vegetables of Peshawar, Pakistan. RSC Adv.

[36] Rashid A, Schutte BJ, Ulery A, Deyholos MK, Sanogo S, Lehnhoff EA, Beck L (2023). Heavy metal contamination in agricultural soil: environmental pollutants affecting crop health. Agronomy.

[37] Devika OS, Singh S, Sarkar D, Barnwal P, Suman J, Rakshit A (2021). Seed priming: a potential supplement in integrated resource management under fragile intensive ecosystems. Front Sustain Food Syst.

[38] Guo J, Liu S, Li X, Liu F: Crop exposure to cold stress: Responses in physiological, biochemical and molecular levels. In: *Sustainable Crop Productivity and Quality Under Climate Change.* Elsevier; 2022: 1–19.

[39] Meena K, Meena N, Meena RN, Choudhary M, Meena S, Kumar S. Role of nanotechnology in organic agriculture. Advances in Resting-state Functional MRI. 2023:343–364.

[40] Mohammadi GR, Amiri F (2010). The effect of priming on seed performance of canola (Brassica napus L.) under drought stress. American-Eurasian J Agric Environ Science.

[41] Salam A, Afridi MS, Javed MA, Saleem A, Hafeez A, Khan AR, Zeeshan M, Ali B, Azhar W, Sumaira. Nano-priming against abiotic stress: a way forward towards sustainable agriculture. Sustainability. 2022;14(22):14880.

[42] Kakar HA, Ullah S, Shah W, Ali B, Satti SZ, Ullah R, Muhammad Z, Eldin SM, Ali I, Alwahibi MS: Seed Priming Modulates Physiological and Agronomic Attributes of Maize (Zea mays L.) under Induced Polyethylene Glycol Osmotic Stress. ACS Omega. 2023. 10.1021/acsomega.3c01715 PMC10308401 37396236

[43] Ali S, Ullah S, Khan MN, Khan WM, Razak SA, Wahab S, Hafeez A, Khan Bangash SA, Poczai P (2022). The effects of osmosis and thermo-priming on salinity stress tolerance in Vigna radiata L. Sustainability.

[44] Zaman SAR, Ozdemir FA (2022). Effect of thermopriming and alpha-tocopherol spray in triticum aestivum L. under induced drought stress: a future perspective of climate change in the region. Sains Malaysiana.

[45] Shahrasbi S, Pirasteh-Anosheh H, Emam Y, Ozturk M, Altay V (2021). Elucidating some physiological mechanisms of salt tolerance in Brassica napus L. seedlings induced by seed priming with plant growth regulators. Pak J Bot.

[46] Ibrahim EA (2016). Seed priming to alleviate salinity stress in germinating seeds. J Plant Physiol.

[47] Sh SM (2014). Role of ascorbic acid and α tocopherol in alleviating salinity stress on flax plant (Linum usitatissimum L.). J Stress Physiol Biochem.

[48] Sadiq M, Akram NA, Ashraf M (2017). Foliar applications of alpha-tocopherol improves the composition of fresh pods of Vigna radiata subjected to water deficiency. Turkish J Botany.

[49] Ahmad I, Ullah S, Nafees M (2020). Effect of osmopriming and thermopriming on amelioration of mercuric chloride stress tolerance in mungbean (Vigna radiata L.). Plant Phys Rep.

[50] Kamran M, Malik Z, Parveen A, Huang L, Riaz M, Bashir S, Mustafa A, Abbasi GH, Xue B, Ali U (2020). Ameliorative effects of biochar on rapeseed (Brassica napus L.) growth and heavy metal immobilization in soil irrigated with untreated wastewater. J Plant Growth Regul.

[51] Zhai Y, Yu K, Cai S, Hu L, Amoo O, Xu L, Yang Y, Ma B, Jiao Y, Zhang C (2020). Targeted mutagenesis of BnTT8 homologs controls yellow seed coat development for effective oil production in Brassica napus L.. Plant Biotechnol J.

[52] Khan R, Khan MN, Ullah H, Basit A, Razzaq A, Ahmad M, Ozdemir F (2018). A comparative assessment of proximate and elemental composition six weedy grasses for their potential use as fodder. Prog Nutr.

[53] Naz R, Khan M, Hafeez A, Fazil M, Khan M, Ali B, Javed M, Imran M, Shati A, Alfaifi M. Assessment of phytoremediation potential of native plant species naturally growing in a heavy metal-polluted industrial soils. Brazilian J Biol. 2022;84. 10.1590/1519-6984.264473 36169410

[54] Saeed S, Ullah A, Ullah S, Noor J, Ali B, Khan MN, Hashem M, Mostafa YS, Alamri S (2022). Validating the impact of water potential and temperature on seed germination of wheat (Triticum aestivum L.) via hydrothermal time model. Life.

[55] Makhaye G, Aremu AO, Gerrano AS, Tesfay S, Du Plooy CP, Amoo SO (2021). Biopriming with seaweed extract and microbial-based commercial biostimulants influences seed germination of five Abelmoschus esculentus genotypes. Plants.

[56] Babar BH, Cheema MA, Saleem MF, Wahid A. Screening of maize hybrids for enhancing emergence and growth parameters at different soil moisture regimes. Soil Environ. 2014;33(1).

[57] Javed T, Afzal I, Mauro RP (2021). Seed coating in direct seeded rice: an innovative and sustainable approach to enhance grain yield and weed management under submerged conditions. Sustainability.

[58] Hakim M, Juraimi A, Begum M, Hanafi M, Ismail MR, Selamat A (2010). Effect of salt stress on germination and early seedling growth of rice (Oryza sativa L.). Afr J Biotechnol.

[59] Al-Ansari F, Ksiksi T. A quantitative assessment of germination parameters: the case of Crotalaria persica and Tephrosia apollinea. Open Ecol J. 2016;9(1).

[60] Shah W, Ullah S, Ali S, Idrees M, Khan MN, Ali K, Khan A, Ali M, Younas F (2021). Effect of exogenous alpha-tocopherol on physio-biochemical attributes and agronomic performance of lentil (Lens culinaris Medik.) under drought stress. PLoS ONE.

[61] Chuyong GB, Acidri T (2017). Light and moisture levels affect growth and physiological parameters differently in Faidherbia albida (Delile) A. Chev. seedlings. Acta Physiol Plant.

[62] Hatami M: Stimulatory and inhibitory effects of nanoparticulates on seed germination and seedling vigor indices. Nanoscience and plant–soil systems. 2017:357–385.

[63] Ullah S, Afzal I, Shumaila S, Shah W (2021). Effect of naphthyl acetic acid foliar spray on the physiological mechanism of drought stress tolerance in maize (Zea Mays L.). Plant Stress.

[64] Shah W, Zaman N, Ullah S, Nafees M (2022). Calcium chloride enhances growth and physio-biochemical performance of barley (Hordeum vulgare L.) under drought-induced stress regimes: a future perspective of climate change in the region. J Water Climate Change.

[65] Ahmadi B, Shirani Rad A, Delkhosh B (2014). Evaluation of plant densities on analysis of growth indices in two canola forage (Brassica napus L.). Eur J Exp Biol.

[66] Kim Y-N, Khan MA, Kang S-M, Hamayun M, Lee I-J (2020). Enhancement of drought-stress tolerance of Brassica oleracea var. italica L. by newly isolated Variovorax sp. YNA59. J Microbiol Biotechnol.

[67] Zhang Z, Huang R (2013). Analysis of malondialdehyde, chlorophyll proline, soluble sugar, and glutathione content in Arabidopsis seedling. Bio-Protoc.

[68] Marcińska I, Czyczyło-Mysza I, Skrzypek E, Filek M, Grzesiak S, Grzesiak MT, Janowiak F, Hura T, Dziurka M, Dziurka K (2013). Impact of osmotic stress on physiological and biochemical characteristics in drought-susceptible and drought-resistant wheat genotypes. Acta Physiol Plant.

[69] Muhammad ZI, Maria KS, Mohammad A, Muhammad S, Zia-ur-Rehman F, Muhammad K (2015). Effect of mercury on seed germination and seedling growth of Mungbean (Vigna radiata (L.) Wilczek). J Appl Sci Environ Manage.

[70] Qadir S, Hameed A, Nisa N, Azooz M, Wani MR, Hasannuzaman M, Kazi AG, Ahmad P (2014). Brassicas: responses and tolerance to heavy metal stress. Improv Crops Era Climatic Changes Vol.

[71] Ameen F, Mumtaz S, Ali B, Hussain I, Hafeez A, Gul A, Elsharkawy MM, Hashim TA, Yasin G, Khan MN. The impact of Cu-polluted and organic soil on the fibrous plant; insights into plant growth promotion, antioxidant defences system, and oxidative stress. Functional Plant Biol. 2023. 10.1071/FP23027 37231613

[72] Daud M, Sun Y, Dawood M, Hayat Y, Variath M, Wu Y-X, Mishkat U, Najeeb U, Zhu S (2009). Cadmium-induced functional and ultrastructural alterations in roots of two transgenic cotton cultivars. J Hazard Mater.

[73] Abeed AH, Saleem MH, Asghar MA, Mumtaz S, Ameer A, Ali B, Alwahibi MS, Elshikh MS, Ercisli S, Elsharkawy MM: Ameliorative Effects of Exogenous Potassium Nitrate on Antioxidant Defense System and Mineral Nutrient Uptake in Radish (Raphanus sativus L.) under Salinity Stress. ACS Omega. 2023. 10.1021/acsomega.3c01039 PMC10308581 37396242

[74] Abeed AH, AL-Huqail AA, Albalawi S, Alghamdi SA, Ali B, Alghanem SM, Al-Haithloul HAS, Amro A, Tammam SA, El-Mahdy M. Calcium nanoparticles mitigate severe salt stress in Solanum lycopersicon by instigating the antioxidant defense system and renovating the protein profile. S Afr J Botany. 2023;161:36-52.

[75] Iqbal B, Hussain F, Khan MS, Iqbal T, Shah W, Ali B, Al Syaad KM, Ercisli S (2023). Physiology of gamma-aminobutyric acid treated Capsicum annuum L.(Sweet pepper) under induced drought stress. PLoS ONE.

[76] Ullah S, Khan MI, Khan MN, Ali U, Ali B, Iqbal R, Z Gaafar A-R, AlMunqedhi BM, Razak SA, Kaplan A. Efficacy of Naphthyl Acetic Acid Foliar Spray in Moderating Drought Effects on the Morphological and Physiological Traits of Maize Plants (Zea mays L.). ACS omega. 2023. 10.1021/acsomega.3c00753 PMC10268277 37323381

[77] Alsherif EA, YaghoubiKhanghahi M, Crecchio C, Korany SM, Sobrinho RL, AbdElgawad H (2023). Understanding the Active Mechanisms of Plant (Sesuvium portulacastrum L.) against Heavy Metal Toxicity. Plants.

[78] Zhang T, Lu Q, Su C, Yang Y, Hu D, Xu Q (2017). Mercury induced oxidative stress, DNA damage, and activation of antioxidative system and Hsp70 induction in duckweed (Lemna minor). Ecotoxicol Environ Saf.

[79] Skudra I, Ruza A (2017). Effect of nitrogen and sulphur fertilization on chlorophyll content in winter wheat. Rural Sustain Res.

[80] Rehman SU, Bilal M, Rana RM, Tahir MN, Shah MKN, Ayalew H, Yan G (2016). Cell membrane stability and chlorophyll content variation in wheat (Triticum aestivum) genotypes under conditions of heat and drought. Crop Pasture Sci.

[81] Arjenaki FG, Jabbari R, Morshedi A (2012). Evaluation of drought stress on relative water content, chlorophyll content and mineral elements of wheat (Triticum aestivum L.) varieties. Int J Agric Crop Sci.

[82] Saleem A, Zulfiqar A, Saleem MZ, Ali B, Saleem MH, Ali S, Tufekci ED, Tufekci AR, Rahimi M, Mostafa RM (2023). Alkaline and acidic soil constraints on iron accumulation by Rice cultivars in relation to several physio-biochemical parameters. BMC Plant Biol.

[83] Alwutayd KM, Alghanem SMS, Alwutayd R, Alghamdi SA, Alabdallah NM, Al-Qthanin RN, Sarfraz W, Khalid N, Naeem N, Ali B. Mitigating chromium toxicity in rice (Oryza sativa L.) via ABA and 6-BAP: Unveiling synergistic benefits on morphophysiological traits and ASA-GSH cycle. Sci Total Environ. 2023:168208. 10.1016/j.scitotenv.2023.168208 37914115

[84] Faryal S, Ullah R, Khan MN, Ali B, Hafeez A, Jaremko M, Qureshi KA (2022). Thiourea-capped nanoapatites amplify osmotic stress tolerance in Zea mays L. by conserving photosynthetic pigments, osmolytes biosynthesis and antioxidant biosystems. Molecules.

[85] Beni AA, Esmaeili A (2020). Biosorption, an efficient method for removing heavy metals from industrial effluents: a review. Environ Technol Innov.

[86] Mwamba T, Islam F, Ali B, Lwalaba J, Gill R, Zhang F, Farooq M, Ali S, Ulhassan Z, Huang Q (2020). Comparative metabolomic responses of low-and high-cadmium accumulating genotypes reveal the cadmium adaptive mechanism in Brassica napus. Chemosphere.

[87] Sabir MA, Nawaz MF, Khan TH, Zulfiqar U, Naseer J, Hussain S, Gul S, Maqsood MF, Iqbal R, Ali B (2023). Impact of dust load and lead (Pb) stress on leaf functioning of urban vegetation. Turk J Agric For.

[88] Elbaz A, Wei YY, Meng Q, Zheng Q, Yang ZM (2010). Mercury-induced oxidative stress and impact on antioxidant enzymes in Chlamydomonas reinhardtii. Ecotoxicology.

[89] Pravisya P, Jayaram K (2015). Priming of Abelmoschus esculentus (L.) Moench (okra) seeds with liquid phosphobacterium: an approach to mitigate drought stress. Trop Plant Res.

[90] Javed J, Rauf M, Arif M, Hamayun M, Gul H, Ud-Din A, Ud-Din J, Sohail M, Rahman MM, Lee I-J (2022). Endophytic fungal consortia enhance basal drought-tolerance in Moringa oleifera by upregulating the antioxidant enzyme (APX) through Heat shock factors. Antioxidants.

[91] Ali B, Wang X, Saleem MH, Azeem MA, Afridi MS, Nadeem M, Ghazal M, Batool T, Qayyum A, Alatawi A (2022). Bacillus mycoides PM35 reinforces photosynthetic efficiency, antioxidant defense, expression of stress-responsive genes, and ameliorates the effects of salinity stress in maize. Life.

[92] Ali B, Wang X, Saleem MH, Sumaira, Hafeez A, Afridi MS, Khan S, Ullah I, Amaral Júnior ATd, Alatawi A. PGPR-mediated salt tolerance in maize by modulating plant physiology, antioxidant defense, compatible solutes accumulation and bio-surfactant producing genes. Plants. 2022;11(3):345. 10.3390/plants11030345 PMC8840115 35161325

[93] Ali B, Hafeez A, Afridi MS, Javed MA, Sumaira, Suleman F, Nadeem M, Ali S, Alwahibi MS, Elshikh MS. Bacterial-Mediated Salinity Stress Tolerance in Maize (Zea mays L.): A Fortunate Way toward Sustainable Agriculture. ACS omega. 2023. 10.1021/acsomega.3c00723 PMC10275368 37332827

