# Correction: Mitigation effect of alpha-tocopherol and thermo-priming in *Brassica napus* L. under induced mercuric chloride stress

**DOI:** 10.1186/s12870-024-04838-7

**Published:** 2024-02-29

**Authors:** Fazal Amin, Arwa Abdulkreem AL-Huqail, Sami Ullah, Muhammad Nauman Khan, Alevcan Kaplan, Baber Ali, Majid Iqbal, Fahmy Gad Elsaid, Sezai Ercisli, Tabarak Malik, Sami Asir Al-Robai, Amany H. A. Abeed

**Affiliations:** 1 Department of Botany, University of Peshawar, Peshawar, 25120 Pakistan; 2 Department of Biology, College of Science, Princess Nourah bint Abdulrahman University, P.O. Box 84428, Riyadh, 11671 Saudi Arabia; 3 Department of Botany, Islamia College, Peshawar, 25120 Pakistan; 4 Biology Laboratory, University Public School, University of Peshawar, Peshawar, 25120 Pakistan; 5 Department of Crop and Animal Production, Sason Vocational School, Batman University, Batman, 72060 Turkey; 6 Department of Plant Sciences, Quaid-I-Azam University, Islamabad, 45320 Pakistan; 7 Institute of Geographic Sciences and Natural Resources Research, University of Chinese Academy of Sciences, Beijing, 100040 China; 8 Biology Department, College of Science, King Khalid University, Abha, Al-Faraa, Asir, 61421 Saudi Arabia; 9 Department of Horticulture, Agricultural Faculty, Ataturk University, Erzurum, 25240 Turkey; 10 Department of Biomedical Sciences, Institute of Health, Jimma University, P.O. BOX 378, Jimma, Ethiopia; 11 Department of Biology, Faculty of Science, Al-Baha University, Al-Baha, 1988 Saudi Arabia; 12 Department of Botany and Microbiology, Faculty of Science, Assiut University, Assiut, 71516 Egypt

Correction: BMC Plant Biol **24**, 108 (2024)

10.1186/s12870-024-04767-5

Following publication of the original article [1], author would like to update Arwa Abdulkreem AL-Huqail’s affiliation. The updated affiliation is given below and the changes have been highlighted in **bold typeface**.

Original affiliation:

Department of Biology, College of Science, Princess Nourah bint Abdulrahman University, Riyadh, 11671, Saudi Arabia

Updated affiliation:

Department of Biology, College of Science, Princess Nourah bint Abdulrahman University, **P.O. Box 84428**, Riyadh, 11671, Saudi Arabia

The correction does not affect the overall result or conclusion of the article. The original article has been corrected.
